# Towards Fluorescence *In Vivo* Hybridization (FIVH) Detection of *H*. *pylori* in Gastric Mucosa Using Advanced LNA Probes

**DOI:** 10.1371/journal.pone.0125494

**Published:** 2015-04-27

**Authors:** Sílvia Fontenete, Marina Leite, Nuno Guimarães, Pedro Madureira, Rui Manuel Ferreira, Céu Figueiredo, Jesper Wengel, Nuno Filipe Azevedo

**Affiliations:** 1 LEPABE, Laboratory for Process Engineering, Environment, Biotechnology and Energy, Faculty of Engineering, University of Porto, Porto, Portugal; 2 Instituto de Investigação e Inovação em Saúde, Universidade do Porto, Porto, Portugal; 3 IPATIMUP, Institute of Molecular Pathology and Immunology of the University of Porto, Porto, Portugal; 4 Nucleic Acid Center, Department of Physics, Chemistry and Pharmacy, University of Southern Denmark, Odense M, Denmark; 5 ICBAS, Institute of Biomedical Sciences Abel Salazar, University of Porto, Porto, Portugal; 6 IBMC, Institute for Molecular Biology and Cell Biology, Porto, Portugal; 7 FMUP, Faculty of Medicine of Porto University, Porto, Portugal; Institut Pasteur Paris, FRANCE

## Abstract

In recent years, there have been several attempts to improve the diagnosis of infection caused by *Helicobacter pylori*. Fluorescence *in situ* hybridization (FISH) is a commonly used technique to detect *H*. *pylori* infection but it requires biopsies from the stomach. Thus, the development of an *in vivo* FISH-based method (FIVH) that directly detects and allows the visualization of the bacterium within the human body would significantly reduce the time of analysis, allowing the diagnosis to be performed during endoscopy. In a previous study we designed and synthesized a phosphorothioate locked nucleic acid (LNA)/ 2’ O-methyl RNA (2’OMe) probe using standard phosphoramidite chemistry and FISH hybridization was then successfully performed both on adhered and suspended bacteria at 37°C. In this work we simplified, shortened and adapted FISH to work at gastric pH values, meaning that the hybridization step now takes only 30 minutes and, in addition to the buffer, uses only urea and probe at non-toxic concentrations. Importantly, the sensitivity and specificity of the FISH method was maintained in the range of conditions tested, even at low stringency conditions (e.g., low pH). In conclusion, this methodology is a promising approach that might be used *in vivo* in the future in combination with a confocal laser endomicroscope for *H*. *pylori* visualization.

## Introduction

Microbial communities coexisting within the human host are known as the human microbiome. Under normal circumstances, there is a very high number of microorganisms that protect the human body, however the shift from a normal to an abnormal microbiome may predispose the individual to several diseases [1]. *Helicobacter pylori* is part of the gram-negative bacterial flora that colonizes the human gastric mucosa and establishes a persistent infection [2]. *H*. *pylori* causes chronic gastritis, which may progress to gastric or duodenal ulcers, gastric atrophy, mucosa-associated lymphoid tissue lymphoma and adenocarcinoma [3]. This bacterium is thus one of the most important pathogens and is responsible for at least half a millions deaths per year [4]. *H*. *pylori* can be detected by invasive methods which include endoscopy, where a biopsy is removed and further used for histopathological examination, rapid urease test, PCR or bacterial culturing [5,6]. High-definition endoscopy offers a potential to improve diagnostic accuracy, allowing a real-time decision-making. Recently, a new endoscope imaging technology was developed, the confocal laser endomicroscopy [7], enabling real time *in vivo* detection during ongoing endoscopy [8,9]. This equipment is based on the excitation of a fluorochrome through a laser which provides a clear two-dimensional image of the tissue [10,11]. It includes a powerful microscope that allows clinicians to view bacteria in real time and contains a water jet nozzle which could be used to inject a solution into the gastric mucosa [12]. Confocal laser endomicroscopy can be in theory combined with fluorescent *in situ* hybridization (FISH) for the identification of microorganisms. The high sensitivity and specificity of FISH and the speed at which the assays can be performed have made FISH an important methodology in clinical microbiology [13,14]. Another advantage is the direct visualization of the bacterium within the sample[15]. Because the fluorescent signal generated by FISH has a strong signal-to-noise ratio, automated image analysis is possible to be implemented [16]. However, the development of FISH methods allowing the detection of specific microorganism in living cells (called FIVH, fluorescence *in vivo* hybridization) has proved to be a challenge [17] as many technical issues have to be overcome. For instance, to perform FIVH in the human gastric stomach it would be necessary for the method to work in very acidic conditions. Consequently, only a highly acid-resistant and efficient oligonucleotide will allow such detection. In recent years, a variety of modified oligonucleotides have been developed to increase not only the stability in biological media but also the ability to bind specifically to a target [18]. Oligonucleotides containing locked nucleic acid (LNA) and 2’-*O*-Methyl-RNA (2’OMe) nucleotide monomers are examples of such oligonucleotides. Both LNA and 2’OMe can be considered as RNA mimics and have shown biological stability, lack of detectable toxicity and potent biological activities [18–23]. Many variations of backbone modifications, most notably the phosphorothioate (PS) backbone, have also evolved and even advanced to the level of clinical trials. [24,25]. PS oligonucleotides containing LNA monomers thus improve target affinity and display significant nuclease resistance [26,27].

In a previous work from our group, the use of oligonucleotide probes composed of a mixture of LNA and 2’OMe nucleotides (LNA/2’OMe) with a PS backbone modification were shown to have an excellent performance at 37°C, displaying higher affinity, increased specificity, faster hybridization kinetics, and superior ability to hybridize to the target [17]. However, current FISH protocols have not been optimized for *in vivo* use. Most, if not all, hybridization buffers are toxic and/or require incubation times that are too long to be used in FIVH. Additionally, FISH protocols are performed at physiological pH (pH 7.0–7.5), and no studies are known which analyzed the behavior of oligonucleotides for FISH under acidic pH conditions.

In order to address these questions, herein we have tested the chemical stability and efficiency of hybridization of the HP_ LNA/2OMe _PS oligonucleotide probe in a range of pH values and in the presence of pepsin. We then evaluated the specificity and toxicity of this oligonucleotide in a human gastric epithelial cell line.

## Materials and Methods

### 1. Oligonucleotide synthesis

The sequence of the probe was selected based on the parameters described in our previous study [17] (Table 1). The oligonucleotides were synthesized on an automated DNA synthesizer using standard phosphoramidite chemistry at 1.0 μmole scale. Two different probes were synthesized using the same sequence but with two different fluorescent labels: fluorescein (FAM) and cyanine 3 (Cy3). Oligonucleotides were purified by reverse phase HPLC (RP-HPLC) and characterized by IonExchange HPLC conditions (IE-HPLC) on a Dionex system HPLC (VWR) and matrix-assisted laser desorption ionization time-of-flight mass spectrometry (MALDI-TOF) on a Microflex Maldi (Bruker Instruments, Leipzig, Germany). The purified oligonucleotides were precipitated by acetone and their purity (>90%) and composition was verified by IE-HPLC and MALDI-TOF analysis. The probes were resuspended in different hybridization buffers as described in the following sections.

**Table 1:** Designation and sequence of the oligonucleotide probes containing locked nucleic acid (LNA; with L superscript) and 2’-O-methyl RNA (2’-OMe; in Boldface) nucleotide monomers.

Designation	Sequence (10-mer)
FAM HP_ LNA/2OMe _PS	5'- FAM G^L^ **AC**T^L^ **AA**G^L^ **CC**C^L^-3’
Cy3 HP_ LNA/2OMe _PS	5'- Cy3 G^L^ **AC**T^L^ **AA**G^L^ **CC**C^L^-3’

HP_ LNA/2OMe _PS is a phosphorothioate oligomer (PS backbones) labeled with either FAM (Fluorescein) or Cy3 (Cyanine).

### 2. Analysis of probe integrity at low pH by analytical chemistry

To determine if the integrity of the fluorochrome-labeled HP_ LNA/2OMe _PS oligonucleotide probe was maintained at low pH and in the buffer used in FISH, we exposed the probe to pH 2 and pH 4 (using a 0.5M urea and 900 mM NaCl buffer) in the same buffer for 3 hours at 37°C. To correctly perform the characterization, the salts in the solution were removed using illustra NAP-10 columns (GeHealthcare, UK). Afterwards, whilst the probe was submersed in different buffers, it was characterized by ion exchange HPLC (IE-HPLC) on a Dionex system HPLC (VWR) using a Dionex DNAPac PA-100, 9*x*250mm analytical column, and by matrix-assisted laser desorption ionization time-of-flight mass spectrometry (MALDI-TOF) on a Microflex Maldi (Bruker Instruments, Leipzig, Germany). As a control, the same characterization was performed in parallel for a suspension of the HP_ LNA/2OMe _PS probe that was not exposed to acidic conditions.

### 3. Bacterial strains and culture conditions

All bacterial cultures were grown in trypticase soy agar (TSA) supplemented with 5% (v/v) sheep blood (Becton Dickinson GmbH, Germany) and incubated for 48 hours at 37°C under microaerobic conditions using a GENbox microaer (bioMérieux, Marcy l’Étoile, France). Bacteria were collected from TSA plates using water or saline (microscopy or cytometry analysis, respectively). The bacterial density was determined by the dilution of initial culture in water or saline and the absorbance was measured at 600 nm. *H*. *pylori* 26695 was used for the optimization of probe hybridization conditions, whereas other bacteria, either *Helicobacter spp*. or the non-*Helicobacter spp*., were used for the analysis of probe specificity and sensitivity. All bacteria used in this study are listed in Table 2.

**Table 2:** *Helicobacter* and non-*Helicobacter* bacterial strains included in this study.

*Helicobacter spp*.		*Non-Helicobacter spp*.
*H*.* pylori* strains	*Non-pylori Helicobacter strains*	
26695 (ATCC 700392)	*H*.* cinaedi* 33221–1.2^b^	*Staphylococcus epidermidis* (ATCC RP602A^**)**^
G27 (NCTC 13282)	*H*.* mustelae* 2H1^b^	*Staphylococcus* aureus (ATCC 25923^**)**^
60190 (*ATCC* 49503)	*H*.* salomonis* ^b^	*Pseudomonas fluorescens* (ATCC 13525^**)**^
84-183(ATCC 53726)	*H*.* muridarum 2*A5+^c^	*Escherichia coli* (CECT 434)
*H*.* pylori* CI-31^a^	*H*.* pametensis* ^c^	*Campylobacter jejuni*
*H*.* pylori* CI-116^a^	*H*.* bilis* ^b^	*Campylobacter coli*
	*H*.* canis* CIP104753	
	*H*. *canadensis* ^b^	
	*H*. *acinonychis* (ATCC 51101)	

Legend: *Helicobacter* clinical isolates provided by:

^a^ Céu Figueiredo [28];

^b^ Francis Megraud, and

^c^ Jay Solnick

### 4. Analysis of probe behavior in a pH range

In a previous study we showed that the FAM HP_LNA/2OMe_PS probe is able to efficiently detect *H*. *pylori* at the human body temperature (37°C) [17]. Here, we further evaluate if this probe efficiently hybridize under conditions similar to those of the human stomach, particularly the acidic pH. Therefore, the first analysis encompassed the evaluation of the performance of this probe at a range of pH values using different hybridization times applying the FISH protocol in suspension, as previously described [17]. For this analysis we used the response surface methodology (RSM) [29,30] to quantify the relationship between the response (or output variable, i.e. fluorescence intensity upon probe hybridization) and the independent variable (input variable, i.e. pH and time). Having used the central compose design, formulated through the statistical software package Design Expert 9.0.3 (StatEase Inc., Minneapolis, USA), we analyzed the variation of fluorescence intensity as a function of pH change and time of hybridization. This design was built in a quadratic surface, where all experiments were conducted in 14 runs, and in which six of them were center points. Each experiment was carried out in duplicate. The levels of the independent variables in pH *versus* time are presented in Table 3. The levels selected for the time and pH variables (corresponding to -1, 0 and 1 in coded units) were based on the hybridization time described for FISH experiments [17,31,32] and to cover the range of physiological conditions of the human stomach [33], respectively. Therefore, the fluorescence intensity (arbitrary units of fluorescence, AUF) was taken as a response to derive the model.

**Table 3:** Experimental levels of variables tested for fluorescence intensity, using the response surface methodology.

Variables	Range and level				
	-α	-1	0	+1	+α
**Time (min)**	0.07	15.5	52.7	90	105.4
**pH**	0.96	2	4.5	7	8.04

### 5. Optimization of probe hybridization conditions in bacterial suspensions

#### a. Optimization of the washing step

To confirm the results obtained by RSM, we studied the probe’s performance at pH 2, 4 or 7, the three most relevant ranges of pH in the human stomach corresponding to the pH of the lumen, mucus, and epithelial layer, respectively. Moreover, for each pH value we tested different incubation times with washing buffer ranging from 0, 5 and 15 min and different washing buffers. We compared the performance of the probe in the standard FISH washing buffer [pre-warmed solution (pH 10), containing 5 mM Tris Base (Fisher Scientific), 15 mM NaCl (Panreac) and 1% Triton X (Panreac)] and in a non-toxic washing buffer (aqueous solution at the pH under study). All FISH experiments were performed in suspension using the protocol described in a previous work [17], with the exception of the time and pH parameters referred above.

#### b. Optimization of the hybridization step

Since we intend to adapt the FISH methodology to be used *in vivo* directly in the gastric mucosa, we have tested a short hybridization time period aiming to reduce the overall time of the hybridization step, since the standard time currently used is 90 minutes [15,17,31]. Therefore, using the optimized washing step described above we tested the hybridization step at 30 and 90 minutes. Following this, specificity and sensitivity analysis was performed with the reduced time using other *H*. *pylori* 26695 and G27 strains and *Helicobacter spp* (*H*. *cinaedi*, *H*. *mustelae*, *H*. *salomanis*, *H*. *muridarum*, *H*. *pametensis*, *H*. *bilis and H*. *canis*).

We optimized the probe with a buffer containing urea to substitute formamide in a previous study [17]. However, in order to use a completely non-toxic buffer, a larger number of experiments were performed to remove some of the toxic compounds that are normally present in the washing and hybridization buffer, such as dextran sulphate, Triton-X and ethylenediaminetetraacetate (EDTA) disodium salt 2-hydrate. Therefore, we tested a hybridization buffer containing 4M urea (VWR BHD Prolabo, Haasrode, Belgium), 900 mM NaCl (Panreac) and different buffer solutions to keep the desired pH (pH2:KCl-HCl, pH4:phosphate-citrate, pH7:Tris-HCl buffer). The following procedure aimed at reducing the concentration of urea and we therefore tested 2M and 0.5M of urea in the same conditions. Thus, in the final optimized protocol, 100 μL of fixed cells (50% ethanol during 15 minutes) were resuspended in 100 μL of hybridization solution (0.5M of urea, 900 mM NaCl and pH buffer solution) with 400 nM probe, and the resulting mixture was incubated at 37°C for 30 min. After hybridization, samples were centrifuged at 14.000 rpm for 5 min, resuspended in 500 μL of washing solution (buffer solution diluted in MiliQ water) and incubated at 37°C for 15 min. The cells were again centrifuged at 14 000 rpm for 5 min and resuspended in 100 μL of saline. To remove aggregates, samples were filtered by a sterile filter (0.22 μm filter, Frilabo). A specificity and sensitivity analysis of the probe in optimized conditions was performed using other *H*. *pylori* 26695 and G27 strains and *Helicobacter spp* (as above).

FISH in slides was performed as previously reported in Fontenete *et al*.,2013 [17,34], with a few modifications. For permeabilization on glass slides, smears of each species/strain were immersed in 50% (v/v) ethanol for 10 min and allowed to air dry. The hybridization was performed using 20 μl of hybridization buffer with 200 nM probe, such that the resulting mixture was covering each smear individually. Samples were covered with coverslips and incubated for 30 min at 37°C. Slides were subsequently washed in a preheated aqueous acid solution for 15 min at 37°C whereupon the slides were allowed to air dry. All experiments were performed in triplicate and for each experiment a negative control (same hybridization conditions, but without a probe in the hybridization mixture) was included. Slides were stored in the dark before microscopy analysis.

#### c. Optimization of the permeabilization step

Most FISH protocols include a fixative/permeabilization step where toxic compounds such as paraformaldehyde (PF) are used. Since PF is toxic for cells, we compared the efficiency of probe hybridization under conditions with (normal protocol) and without PF in the FISH protocol.

#### d. Optimization of FISH protocol in conditions similar to gastric juice

Since human gastric mucosa is covered by gastric juice, the washing step in FIVH could be considered the gastric juice. Therefore we substituted the standard washing buffer in the FISH protocol by a gastric simulated juice that contains pepsin [35]. The hybridization step was performed in *H*. *pylori* 26695 bacterial suspension, using 0.5M urea buffer at different pH values (2, 4 and 7). Afterwards specificity and sensitivity analysis was performed with the bacteria described in Table 2.

### 5. Cytometry analysis

*H*. *pylori* bacterial cell suspensions stained with Cy3-labeled or FAM-labeled HP_ LNA/2OMe _PS oligonucleotide probes, and the respective unstained negative controls, were analyzed in a Beckman Coulter Epics XL flow cytometer (Brea, USA) equipped with a 488 nm laser. For each sample, 20 000 events were collected. All the experiments were repeated in triplicate.

### 6. Microscope evaluation

Bacteria images were acquired with a Carl Zeiss Apotome Axiovert 200M Fluorescence Microscope (Carl Zeiss, Jena, Germany). Images were taken with an Axiocam HRm camera and processed with Zeiss Axion Vision 4.8 software. All the experiments were performed in triplicate.

AGS (gastric adenocarcinoma) cells were analyzed on an inverted epi-fluorescence microscope, (Axiovert 200M, Zeiss,Germany). Images were acquired with a Leica TCP SP2 AOBS camera and processed LAS AF using Lite software (Leica Microsystems CMS GmbH).

### 7. Cell culture conditions and infection with *H*. *pylori*

Human gastric epithelial cell line AGS (ATCC CRL-1739) was maintained at 37°C under 5% CO_2_ humidified air, in RPMI medium 1640 Glutamax I (Gibco, Invitrogen, Grand Island, NY, USA) supplemented with 10% (v/v) fetal bovine serum (FBS) and 1% (v/v) Penicillin/Streptomycin (complete medium). Culture medium was replaced every two days.

For infection experiments, AGS cells were seeded in a 6-well culture plate and grown in antibiotic-free medium until reach the desired confluence. AGS cells were infected with *H*. *pylori* at a multiplicity of infection (MOI) of 100, for 3 hours. Controls without infection were also seeded in the same conditions. In order to evaluate the specificity of Cy3 HP_ LNA/2OMe _PS, FISH was performed with different concentrations of probe and the fluorescence signal was studied by confocal microscopy.

### 8. Cell proliferation assay

AGS cells were seeded in 96-well plates with a final volume of 200 μL of complete medium per well and grown to 50% and 100% confluence. Upon reaching the desired confluence, AGS cells were incubated with 200 nM of Cy3_ HP_ LNA/2OMe _PS diluted in hybridization buffer [0.5 M urea and 900 mM NaCl; 1% (v/v) or 5% (v/v)] or in complete medium (untreated control) for 24 hours. At the end of the incubation period, the cell viability was evaluated using CellTilter 96 Aqueous One Solution Cell Proliferation Assay (Promega Corporation, Madison, WI), according to the manufacturer’s instructions. Absorbance at 490 and 630 nm was measured using a microplate reader (Biotek Instruments Inc. Synergy MX, USA). Background absorbance values were subtracted from the absorbance values generated with MTS assay. The values from treated cells were compared with the values generated from untreated control cells and reported as percent viability. All experiments were performed in triplicate.

### 9. Cell death analysis in AGS cells

The caspase 3/7 activity in AGS cells treated with probe was analyzed using the luminometric Caspase-Glo assay (Promega, Madison USA), according to manufacturer’s instructions. AGS cell were plated in 96 well solid white bottom plates in 200 μL of complete medium. When AGS cells reached 50% confluence, they were treated with 200 nM of Cy3 HP_ LNA/2OMe _PS diluted in a hybridization buffer (vehicle) or complete media (untreated control) and cells were incubated for 24 hours. The plates were then incubated with 100 μL of Caspase-Glo reagent at room temperature for 30 minutes. The luminescence of each sample was measured in a plate-reading luminometer (Biotek Instruments Inc. Synergy MX, USA). The experiments were performed in triplicate and repeated on three separately-initiated cultures.

Evaluation of apoptosis was performing using the Cell Death Detection ELISA kit (Roche Applied Science, Germany) according to the manufacturer’s protocol. The principle of this assay is the detection of mono- and oligonucleosomes in the cell lysates using biotinylated anti-histone and peroxidase-coupled anti-DNA antibodies. The amount of peroxidase retained in the immunocomplex was photometrically determined with 2,2′-azino-bis- (3-ethylbenzthiazoline-6-sulfonic acid) as the substrate through the absorbance quantification quantified at 405 nm. Data are expressed as mean of three independent experiments. In a separate experiment, AGS (4 x10^4^ cells/ml) were treated for 24 h with 200 nM of Cy3_HP_ LNA/2OMe _PS or vehicle (hybridization buffer)[in 1% (v/v) or in 5% (v/v)]. Wells with serum free medium were used as negative controls. Briefly, cells were lysed by adding lysis buffer to each well and incubating for 30 min at 20°C, 300 rpm. Each plate was centrifuged at 200 x *g* for 10 min, and 20 μL of each supernatant was transferred to streptavidin-coated wells. The wells were treated with anti-histone and anti-DNA-containing immune-reagent, incubated for 2h at 25°C, 250 rpm, washed three times, and treated with peroxidase substrate 2,2’-azino-di-(3-ethyl-benzthiazoline sulfonate). Absorption at 405 nm and 490 nm was measured using a spectrophotometer (Biotek Instruments Inc. Synergy MX, USA). All experiments were repeated three times.

### 10. Statistical analysis

Statistical significance was determined by One-way analysis of variance (ANOVA) by applying the Tukey multiple-comparisons test, using SPSS statistics 17.0 (SPSS, Statistical Package for the Social Sciences, Chicago, USA). Results were expressed as mean values±SD. Differences were considered to be statistically significant when *p*<0.05.

## Results

### 1. Analytical chemistry of HP_LNA/2OMe_PS at low pH and high salt concentrations

Although the most commonly used dye in FISH is FAM, this dye has been described as sensitive to pH [36]. Therefore, we tested the integrity of the probe and fluorochrome conjugate at pH values of 2 and 4. The analytical spectra obtained by IC-HPLC and MALDI showed that most of the FAM-labeled HP_ LNA/2OMe _PS was intact. The spectra obtained by IC-HPLC were very similar in all conditions with identical retention time (~21.8 min) for the oligonucleotide not subjected to acidic conditions and after exposure to pH 2 or pH 4 conditions (S1A Fig). This analytical analysis allowed us to verify the presence of the dye and to confirm the purity of the sample even after exposure to strongly acidic conditions. Similarly, the molar mass of the probe was analyzed and confirmed to be the same under all conditions studied (MALDI-TOF spectrum; S1B Fig). In the mass spectrum of the FAM_HP_ LNA/2OMe _PS, there was no significant change in the molar mass (MW = 4002.003) (S1B Fig). Therefore, the integrity of the probe is maintained at low pH and high salt concentration. The results clearly show that the FAM_ HP_ LNA/2OMe _PS is in principle suitable for applications in the low pH environment of the human stomach.

### 2. Central composite design, pH versus time

In the present study the RSM was employed to identify the interactions between variable pH and hybridization time. Fluorescence intensity values were determined by flow cytometry and corresponded to the average of duplicates. The design matrix and the matching observed responses are shown in S1 Table. The table shows fluorescent intensities corresponding to the combined effect of the studied variables in their specific ranges.

The central composite design with two variables, including six replicates at the central point and one response, was used for fitting a quadratic response surface. A regression analysis was performed to fit the response function with the experimental data. The statistical significance of the linear model equation was checked by ANOVA and the data are shown in S2 Table.

**Fig 1:**
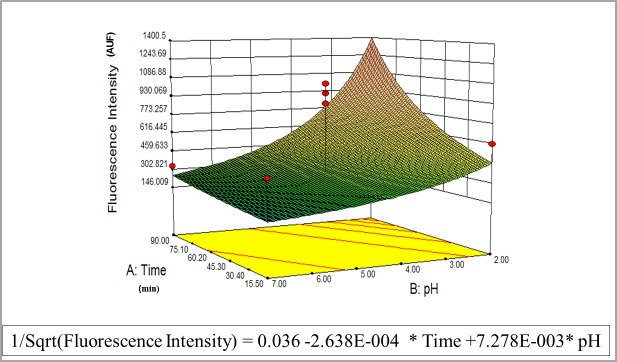
Analysis of FAM HP_LNA/2OMe_PS probe performance using the response surface methodology (RSM). Three dimensional surface plot showing the variation of *H*. *pylori* fluorescence intensity as a function of model terms. The model graphs are represented in gradient color shading. The surface is red at higher response levels and blue at lower ones.

The results obtained were subjected to analysis of variance with the inverse square root model observed in Fig 1. Interaction effects and optimal variable levels were determined by plotting the response surface. The contour plot shows the behavior of response (fluorescence intensity) with respect to simultaneous change in the two variables under study (time and pH).

Although the model was significant for only one of the variables (pH), it helped us understand that the probe could work at a large range of pH values, particularly at low pH. Therefore, after we performed this analysis we carried out experiments without using RSM as a strategy to compare and confirm the model.

### 3. Optimization of washing and probe hybridization conditions

Specificity of probe binding to the target typically depends on the washing conditions. The washing step serves mainly to rinse off excess probe molecules at conditions that prevent unspecific binding [37]. Usually, the washing buffer includes detergents and a salt and the washing procedure takes around 30 minutes. However, in FIVH this type of protocol is not possible; therefore, we tested the use of an aqueous buffer (water with buffer solution) at shorter periods of time (5 to 15 minutes). We observed that it is possible to use aqueous buffer during 15 minutes in the range of pH in study (S2A Fig). A desirable feature of any diagnostic test is that it should achieve results in the shortest possible time. Our alternative approach showed that the hybridization process can be performed in 30 minutes in bacteria, obtaining similar results (S2B Fig).

Several studies use complex hybridization buffers in FISH methodologies containing toxic compounds. Most of them have not been proved to be always necessary to improve the yield of an hybridization reaction involving LNA probes (e.g. EDTA and dextran sulfate). In our experiments at pH 7 we observed no effect in the hybridization efficiency after the elimination of these chemicals from the hybridization solution (S2C Fig). In many FISH protocols, increasing stringency improves specificity with a corresponding loss in sensitivity. The use of denaturants is essential to lower the Tm of the hybrids and increase the stringency of the probe to target binding. Therefore, the use of urea has a crucial role in the hybridization to the target. In our experiments we reduced the quantity of this denaturant from 4 M to 0.5 M which, despite the significant large reduction in concentration, led to no significant decrease observed in fluorescence quantification (S2C Fig).

When performing FIVH the use of toxic compounds such as the fixative buffer is not possible. Consequently, we optimized the permeabilization step by replacing PF for 50% (v/v) ethanol. Unlike in other studies [38,39], no significant differences were observed in the fluorescent signal intensity with this replacement (S2D Fig). We further observed that when the permeabilization step with ethanol was not performed in suspended bacteria, the obtained fluorescence intensity was very weak for all of pH values studied. Nevertheless, in adhered bacteria, high fluorescence was obtained with a non-fixative protocol (S3 Fig).

While acidity profiles in the human stomach can vary extensively [33], the three profiles tested were carefully selected to be representative of the conditions in all groups of patients. We maintained a denaturant, urea, and sodium chloride in the hybridization buffer since these compounds are essential not only for the high efficiency of the FISH methodology but also to adjust the stringency conditions of the hybridization [40].

**Fig 2:**
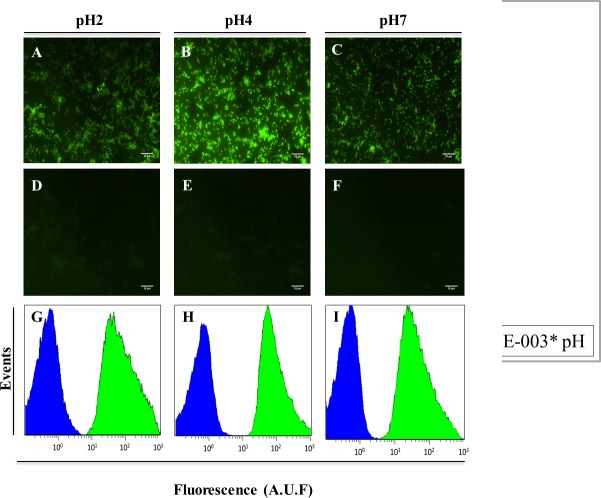
Detection of *H*. *pylori* in slides, by epifluorescent microscopy (A-F), and in suspension, by flow cytometry (H-J), using the FAM HP_ LNA/2OMe _PS oligonucleotides probe at different pH values. A—F Smear of pure culture of *H*. *pylori* strain 26695 observed by epifluorescent microscopy. A-C. Experiment using 200 nM of the probe. D-F. Smears without probe were used as negative control. All images were taken at equal exposure times. G-I. Relative fluorescence histograms of LNA-FISH targeting *H*. *pylori* in different pH for two different assays—Blue: negative control with no probe; Green: positive sample. Scale bar = 10 μm.

After all protocol optimizations, we performed FISH in pure cultures of *H*. *pylori*, in adhered and in suspended bacteria, and evaluation was performed by microscopy (Fig 2A–2F) and flow cytometry (Fig 2G–2I). We tested the probe in adhered bacteria once *H*. *pylori* has colonized the gastric mucosa by adhering to the mucus layer that lining the gastric epithelium [41]. It is already known that adhesion to epithelial cells is essential for the infection step [42]. The FISH performed in adhered bacteria showed to be efficient for all pH values (Fig 2). However, at pH 4 we observed a higher fluorescent intensity. The control without probe showed a low background in the optimized hybridization buffer (Fig 2D–2F).

The results obtained by flow cytometry also showed high fluorescence intensities at all pH values and a low background (Fig 2G–2I). This analysis was also performed using the Cy3-labelled HP_ LNA/2OMe _PS and the results showed significantly brighter staining (S4 Fig) probably due to higher resistance of the fluorochrome Cy3 to photobleaching [40,43].

**Fig 3:**
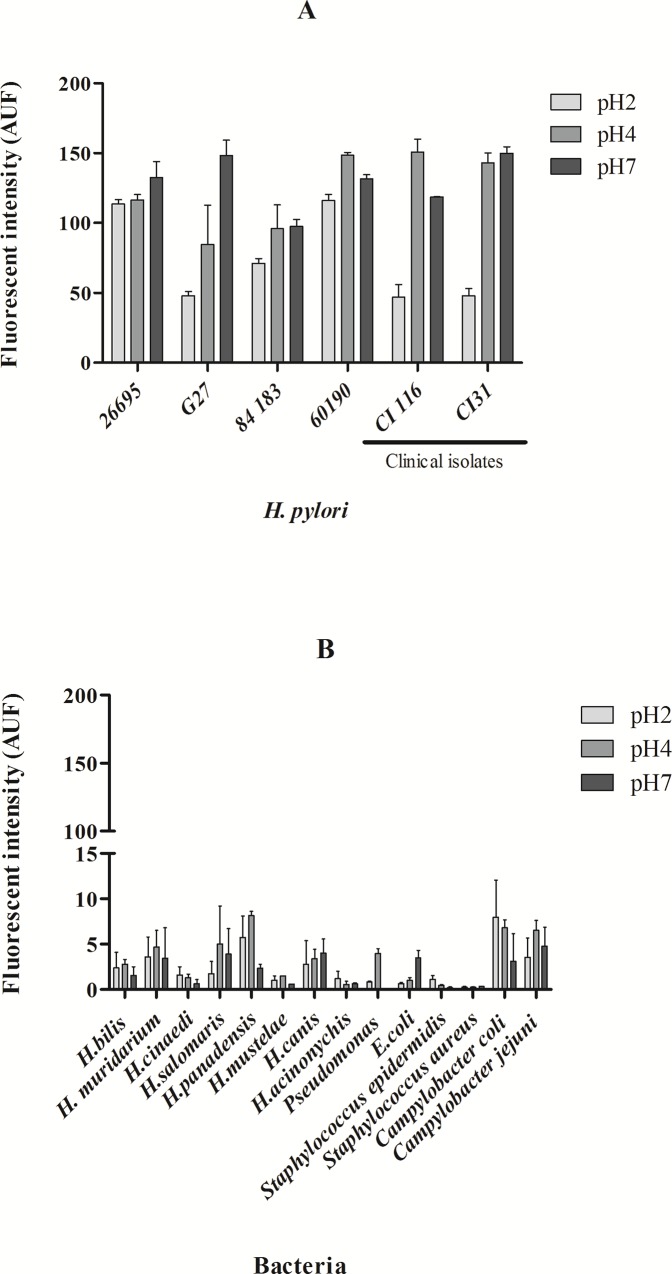
Sensitivity and specificity studies performed with the HP_ LNA/2OMe _PS probe at different pH values evaluated by flow cytometry. A. Sensitivity test using different strains and clinical isolates of *H*. *pylori*. C. Analysis of the probe in clinical isolates of *H*. *pylori*. B. Specificity test using different species of Helicobacter and other bacteria.

The sensitivity studies (Fig 3) were performed with the FAM HP_ LNA/2OMe _PS oligonucleotide probe and showed that this probe is able to detect both *H*. *pylori* reference strains and *H*. *pylori* clinical isolates (Fig 3A), while no fluorescent signal was detected for the non-*pylori Helicobacter* strains tested (Fig 3B). These results also showed that this probe was specific and sensitive for *H*. *pylori* strains, even when the hybridization is carried out under low stringency conditions (low temperature, short hybridization time and low pH).

**Fig 4:**
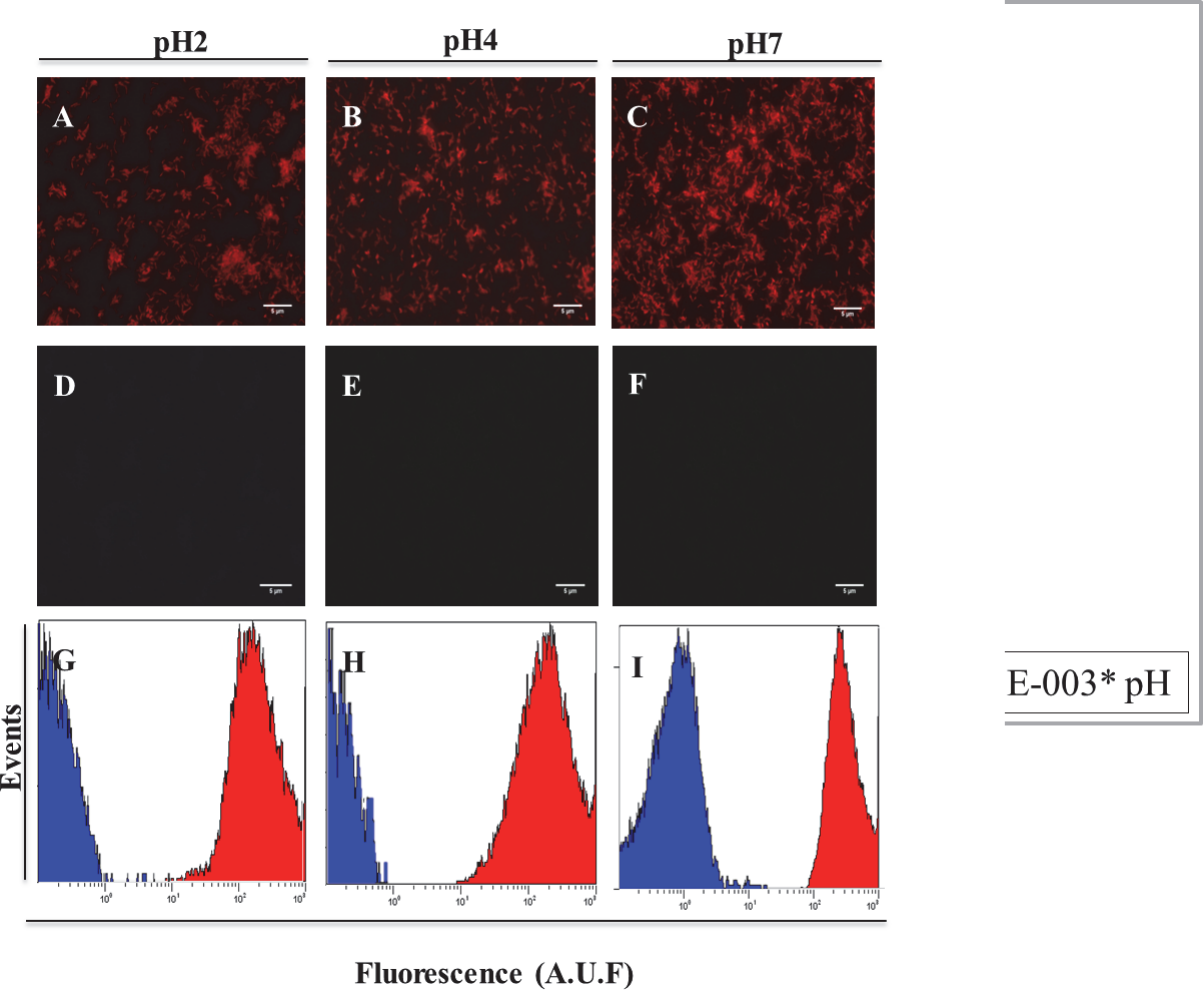
Detection of *H*. *pylori* using the Cy3 HP_ LNA/2OMe _PS oligonucleotide probe in a smears of pure culture of *H*. *pylori* strain 26695 using simulated gastric juice by epifluorescent microscopy. A-F Smear of pure culture of *H*. *pylori* strain 26695 (ATCC 700392) observed by epifluorescent microscopy. A-C Experiment using 200 nM of Cy3 HP_ LNA/2OMe _PS oligonucleotide probe. D-F. Smears without probe were used as negative control. All images were taken at equal exposure times. Original magnification: 1000x. G-I. Relative fluorescence histograms of LNA-FISH targeting *H*. *pylori* in different pH for two different assays—Blue: negative control with no oligonucleotide probe; Red: sample. Scale bar = 5 μm.

**Fig 5:**
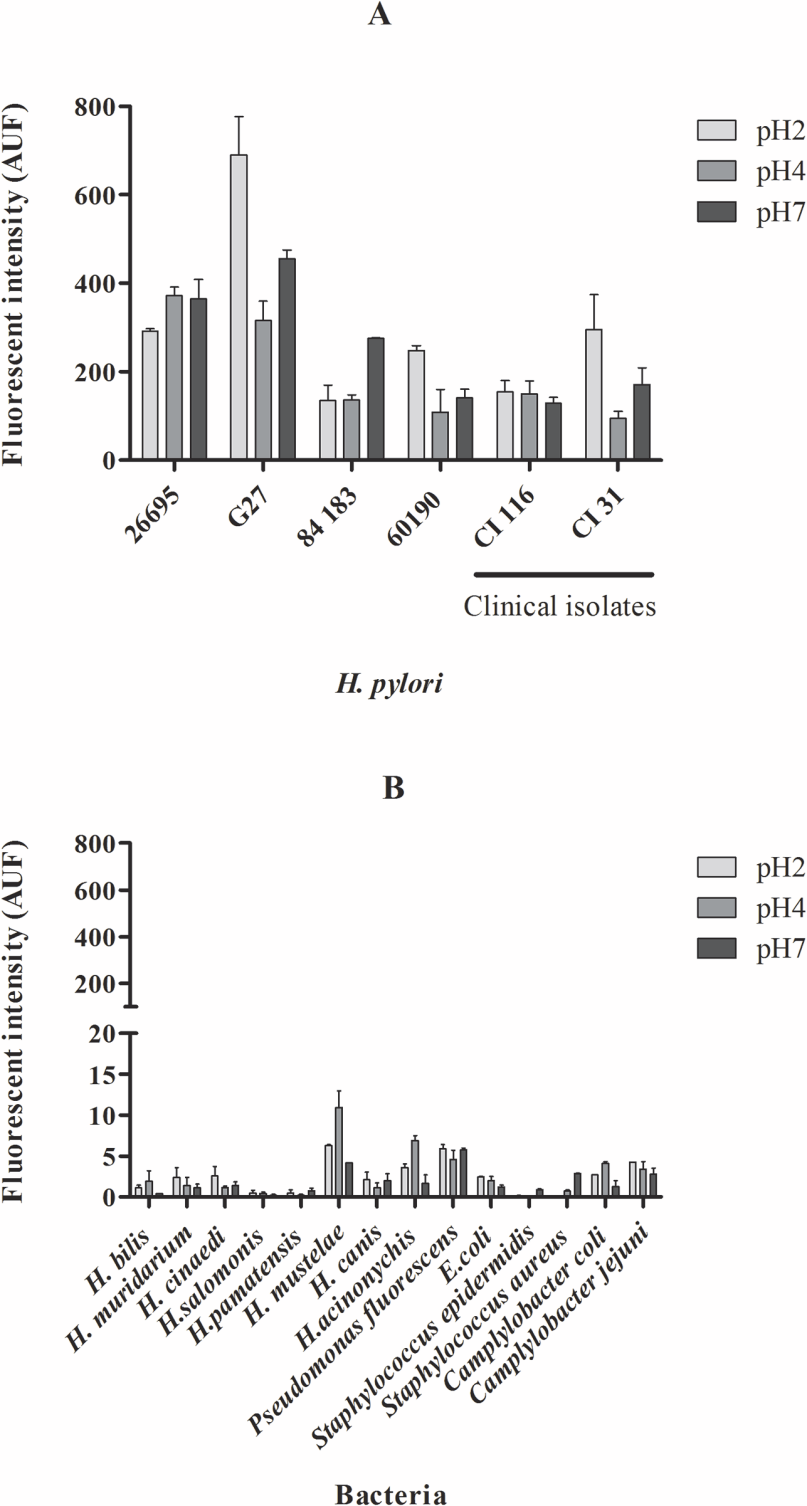
Sensitivity and specificity studies performed with the HP_ LNA/2OMe _PS oligonucleotide probe using simulated gastric juice. A. Sensitivity test using different strains and clinical isolates of *H*. *pylori*. B. Specificity test using different species of Helicobacter and other bacteria.

To mimic the human gastric mucosa, we replaced the water in the washing step of the FISH protocol with simulated gastric juice. We observed that the signal of the FAM_ HP_ LNA/2OMe _PS be was decreased in the presence of this solution (S5 Fig). Therefore, an analytical study to understand if the FAM is functional in this acid solution was performed (S5 Fig). Using this acid solution low fluorescence intensity was observed. A possible explanation is that when the dye oligonucleotide conjugates are subjected to acid conditions, protonation or deprotonation of the dye units can alter their electronic structure which can then affect the ability to fluoresce [44]. A protonation of neighboring nucleobases can also alter their electron-donating properties and therefore determine their quenching abilities with consequent loss of fluorescence [45]. On the other hand, the Cy3-labelled HP_ LNA/2OMe _PS still had a strong signal for all hybridization conditions in the simulated juice (Fig 4). All sensitivity and specificity studies demonstrated that the gastric juice does not interfere with the hybridization efficiency of the Cy3_HP_ LNA/2OMe _PS (Fig 5).

### 4. Detection of *H*. *pylori* in infected gastric AGS cell line

**Fig 6:**
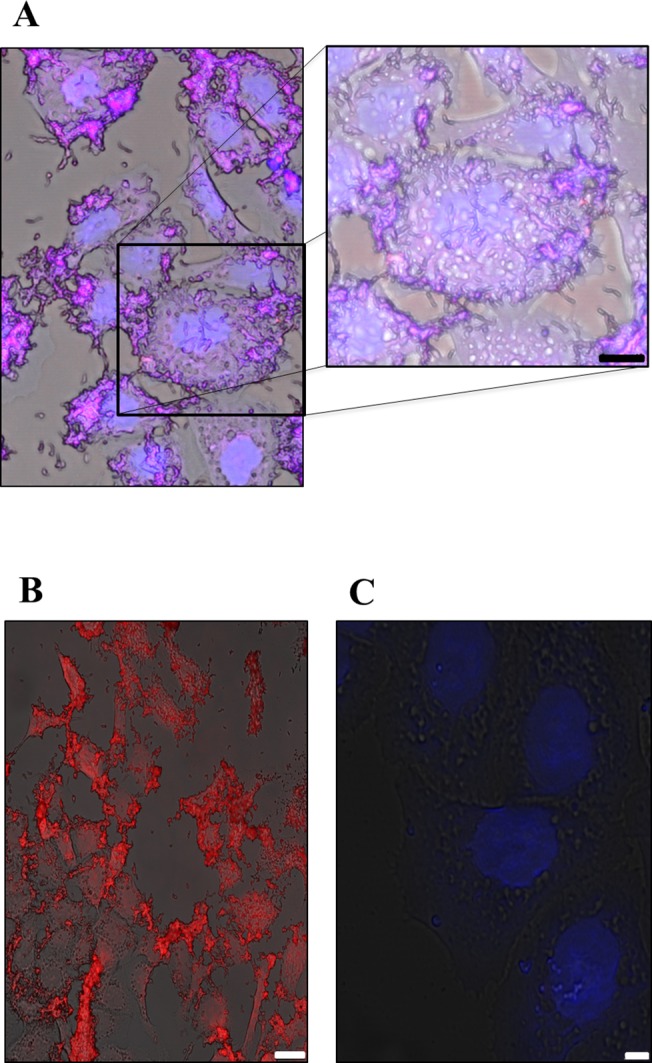
Confocal microscopy images of AGS cells infected with *H*. *pylori* 26695 strain. A. Detection of *H*. *pylori* by the Cy3_ HP_ LNA/2OMe _PS _oligonucleotide probe in infected epithelial cells. Channel red, DAPI and bright field were overlapping. Scale bar = 10μm B. Detection of *H*. *pylori* by Cy3_ HP_ LNA/2OMe _PS _oligonucleotide probe in co-infected cells and isolates bacteria. Channel red, and bright field were overlapping. Scale bar = 25μm C. Uninfected AGS cells stained with Cy3_ HP_ LNA/2OMe _PS _oligonucleotide probe and DAPI. Channel red, and bright field and DAPI were overlapping. Scale bar = 5μm. Red: Cy3 fluorescence. Blue: DAPI staining to counterstain nuclei.

*In vivo H*. *pylori* is found free in the gastric mucus, and also in close contact with epithelial cells [46,47]. A few *in vitro* studies have showed that *H*. *pylori* can be invasive and reside within the cytoplasmic vacuole of the infected cells [48–50]. Therefore, we tested the hybridization of the Cy3_HP_ LNA/2OMe _PS probe in AGS cells infected with *H*. *pylori* by confocal microscopy. The analysis of the confocal showed that the probe can still hybridize to *H*. *pylori* when it is adhered to the gastric cells, and even when a high concentration of bacteria is present (Fig 6A). We also observed a low background of the confocal images obtained (Fig 6B) which is a fundamental feature in this type of studies, since background fluorescence emission is a significant drawback in *in vivo* assays [51]. In these studies, we did not observe any unspecific binding signal as observed in the control samples (only AGS cells) (Fig 6C).

### 5. Toxicity studies

Although LNA modified oligonucleotides offer advantages for improved target specificity they can be hampered by toxicity in non-clinical studies [52,53]. Burdick *et al*. [54] have demonstrated that this toxicity is associated with some sequence motifs. Therefore we have evaluated if the Cy3_HP_ LNA/2OMe _PS induces toxicity to gastric epithelial cells through viability and apoptosis assays.

**Fig 7:**
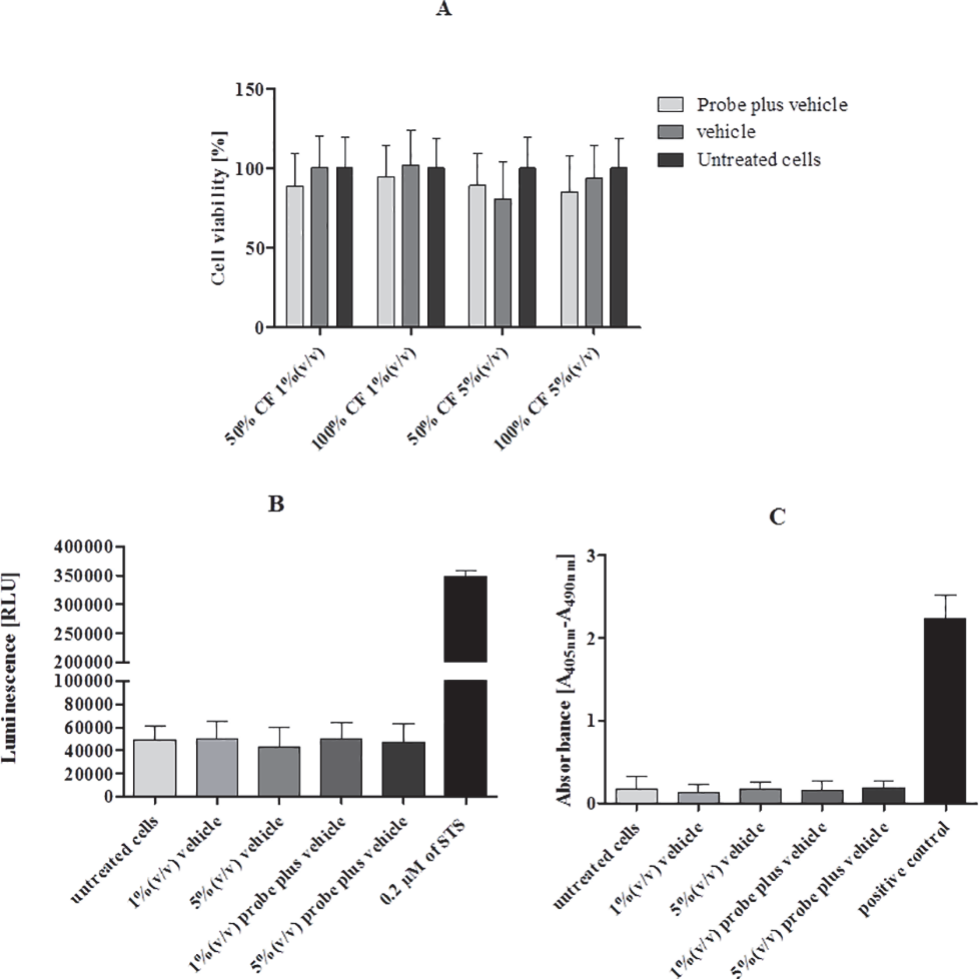
Effect of the Cy3_ HP_ LNA/2OMe _PS oligonucleotide probe on viability (MTS assay, A) and cell death (apoptosis, B and C) of AGS cells. AGS cells were treated with 200 nM of Cy3_ HP_ LNA/2OMe _PS oligonucleotide probe for 24h. The results are the mean ± SEM of three independent experiments; *p<0.*05 vs* untreated cells by ANOVA. A) Cell viability was measured using MTS assay. B) Analysis of caspase-3/7 activity in AGS cells treated with Cy3_ HP_ LNA/2OMe _PS oligonucleotide probe. Staurosporine, STS, was a positive control of cell death. C) Effect of Cy3_ HP_ LNA/2OMe _PS oligonucleotide probe on the amount of DNA fragmentation in the cultured gastric cell line AGS. The level of apoptosis occurring with each treatment was determined by cell death ELISA^Plus^ kit.

The MTS assay was used to investigate the effect of the Cy3_ HP_ LNA/2OMe _PS on AGS cell viability at different confluences (50% and 100%). Treatment of AGS cells with the Cy3_ HP_ LNA/2OMe _PS at 200 nM did not result in a statistically significant decrease in proliferation for any of the confluences (Fig 7A). We also tested the effect of the probe vehicle (hybridization buffer) on cell proliferation, and again no statistically significant differences were observed (p>0.05) relatively to the untreated cells.

Apoptosis is a natural form of cell death that induces condensation of the nucleoplasm and cytoplasm, blebbing of cytoplasmic membranes and fragmentation of the cell into apoptotic bodies that are recognized and eliminated by adjacent cells [55]. Since apoptosis is ultimately mediated by caspase 3 and caspase 7, a Caspase-Glo 3/7 Assay was conducted to determine whether the Cy3_ HP_ LNA/2OMe _PS activates the apoptotic pathway in probe-treated gastric cells. As depicted in Fig 7B, no statistically significant differences regarding activation of caspase 3 and 7 were observed between untreated cells and Cy3_ HP_ LNA/2OMe _PS-treated cells (24 h at 200 nM); the same was found for the vehicle in both volumes tested, which contrasts with staurosporine treatment (positive control for cell death) that induces activation of caspases 3 and 7. The Cell Death Detection^Plus^ system (Roche) was used as another technique to confirm these results (Fig 7C). Cy3_ HP_ LNA/2OMe _PS treatment of AGS cells did not induce cell death, as no significant increase in the ratio of DNA fragmentation was found in comparison to untreated cells. Additionally, no significant differences were observed between the vehicle (hybridization buffer) and untreated cells regarding the induction of DNA fragmentation.

## Discussion

In this study we have tested a LNA probe using a non-toxic and simple FISH protocol as a potential novel technique for *in vivo* diagnosis of *H*. *pylori* infection. The presence of *H*. *pylori* was detected by FISH using *ex vivo* experiments with a specific molecular oligonucleotide probe in conditions that mimic gastric environment. We optimized a fast, simple and non-toxic FISH protocol and analyzed the efficiency, sensitivity and specificity of the probe in a range of pH and using a gastric simulated juice. An *in vitro* analysis of the oligonucleotide probe toxicity in a gastric cell line was also performed. The main objective of this work is to build the knowledge and the necessary tools to detect *H*. *pylori* by FIVH which could provide spatial information on the localization of *H*. *pylori*. Such information is important in the clinic in order to perform a correct and fast diagnosis and to decide the best treatment for the infection.

The pH within the human stomach lumen varies between 1 to neutral after a meal, although in adults the luminal pH rarely exceeds 5.5 [56]. *H*. *pylori* uses this transmucus pH gradient for spatial mobility and orientation to reach zones where the pH is near neutral [57,58]. Therefore pH conditions in the stomach may have a strong effect on the number of *H*. *pylori* cells present in the mucus layers *in vivo*. We initially studied the behavior of the oligonucleotide probe in a range of pH values using RSM as an experiment tool designer. The RSM approach has been used to determine the best conditions for a study comprising two or more variables by calculating the combined effect of selected variables [59,60]. In Fig 1, the response surface shows the effect of the time of hybridization and pH on the fluorescence intensity. The result demonstrates that the response surface has a maximum point at a very low pH and around 50 min of hybridization. However, because the time component was not statistically significant (P>0.05), we can only prove that this probe can work effectively in an acid range of pH values. To the best of our knowledge, this is the first study where FISH is performed under extremely acid conditions. In the majority of the studies, experiments at pH values of 6.5–7.5 or even higher have been applied to obtain more stringent hybridization conditions [15,31].

Conventional hybridization time in FISH performed in bacteria requires 1.5 hours incubation with oligonucleotide probes [15,31]. The decrease of hybridization time has already been described for FISH in mammalian cells or at higher hybridization temperatures [32,61]. Here, we show that a 30 min hybridization step followed by a 15 min washing step could be used to obtain higher fluorescence intensities while maintaining the specificity and sensitivity desired (Figs 3 and 5).

The use of toxic and complex buffers is recurrent in FISH protocols. Although there are some studies which explore novel non-toxic hybridization buffers, some of the toxic reagents have not been removed [32,62,63]. PF is the most commonly used fixative in FISH experiments, however its well described toxicity and carcinogenic proprieties make it impossible to use in FIVH [64]. Some concerns also exist in terms of the effect of the cross-links created by PF with respect to the ability of the probes to recognize their targets [39,65]. In addition, it has been suggested that the RNA target may be degraded during the fixation process [66]. Even though we were able to remove PF from the permeabilization step, the presence of ethanol was shown to be essential for bacteria in suspension (S2D Fig) but not for adhered bacteria (S3 Fig). Because *H*. *pylori* in the human stomach can be found either adhered to the gastric epithelial cells [67] or in suspension in the gastric juice or mucus [68], permeabilization using ethanol was maintained in the protocol.

Other types of toxic compounds currently used in the hybridization buffer in FISH protocols are ion chelators, such as EDTA, which should be used in hybridization experiments with low salt buffers. In the case of high salt conditions, the need of using chelators has not been proven. [69]. It has also been reported that dextran sulfate (the exclusion agent most commonly used in FISH protocols) has only a minor, or no effect at all, in hybridization reactions with short probes [70]. However, the use of denaturant and salt is essential in FISH in order to obtain an efficient and specific hybridization reaction. The replacement of formamide by urea has been reported in some studies [17,62,63]. In our results we showed that the reduction of the concentration of urea in the hybridization solution from 4 M to 0.5 M does not affect the efficiency of the reaction (S2C Fig). The substitution of the salt solution used in the washing step by an aqueous buffer (Fig 2) or gastric juice (Fig 4) also had a positive outcome in the protocol. One possible reason could be the low stringency conditions that we used (low hybridization temperature and high salt concentration). It has generally been accepted that low-stringency hybridization corresponds to a stronger binding of probes to the targeted rRNA sites [71]. Therefore, higher temperatures appear to be less suitable for FISH in terms of obtaining a satisfactory fluorescence signal [38]. However, the absence of an washing step in the reaction leads to the decrease of the oligonucleotide probe specificity [37]. Specificity is one of the most important parameters in FISH methodology and is an essential property of nucleic acids [72]. In our studies we observed that the HP_ LNA/2OMe _PS has higher specificity when used with hybridization buffer (NaCl and urea). Some authors have reported that 2′OMe/LNA PS oligonucleotides have low specificity [73], but in our hands this oligonucleotide probe presented very low or no binding to non-pylori bacteria and no binding to AGS cells under the conditions tested. These differences may be explained by the experimental conditions selected in each study.

Although it is already known that FAM is sensitive to low pH conditions [36,65], we performed a chemical analysis after acid buffer treatments (HCl buffer, pH2 or pH4) and proved that this is not always true. However, when we use simulated gastric juice, our FISH results showed a huge decrease in the fluorescence intensity (S5 Fig). Therefore, we can argue that the fluorescence of this fluorochrome could be rapidly quenched in the presence of pepsin. As a member of the aspartic protease family, pepsin has a significant role in drug research and in pharmacological studies [74], and we therefore can propose that this quenching mechanism could be the key to the decrease of fluorescence of the FAM_2′OMe/LNA PS in simulated gastric juice. The quenching mechanism and the measure of binding constants, binding sites and molecular interaction distances will be investigated in our future work.

It is also known that oligonucleotides can have significant toxic effects in mammalian cells. Some of these effects can be related with certain chemical modifications or with the reagents used as vehicles of probe in the experiments [73]. For the following step in this study, we investigated whether the Cy3_ HP_ LNA/2OMe _PS would induce toxicity in a gastric cell line, AGS, through viability and apoptosis analysis. Although it has been reported, in some studies, that all-phosphorothioate oligonucleotide probes can induce toxicity [75], we observed no significant toxicity under the conditions used (Fig 7). The HP_ LNA/2OMe _PS contains 2’OMe, which is a non-toxic, naturally occurring nucleic acid [76], and therefore it could reduce the toxicity of the oligonucleotide probe. Other authors have reported minimal LNA toxicity in their studies [19,73,77]. Hepatoxicity is also reported in animals for some LNA oligonucleotides [52,78], however, recently, Burdick *et al*., refers that the hepatoxicity of LNA oligonucleotides is associated with specific motifs [54]. LNA-based oligonucleotides have in fact advanced into phase 1 and 2 clinical studies, which also demonstrates the non-toxic character of some sequences [77,79].

In order to establish FIVH in the routine practice of clinical laboratories, several limitations have to be overcome in the future. First, although we reduced the time needed to perform some of the FISH steps, the duration of the entire protocol is still too long. In fact, for FIVH in diagnosis, a further decrease in hybridization time may be beneficial for the patient. Therefore, a new system capable of delivering directly the probe, avoiding the permeabilization step and speeding up the hybridization period, might be useful. Second, FIVH is only possible if confocal laser endomicroscopy is used, and is hence limited to certain areas of the human body. However, despite the difficulties which have to be overcome, our study provides a technological advance not only for current FISH methodologies but also towards the development of FIVH for the diagnosis of pathogens and to assess disturbances in the microbiome.

## Conclusion

In this study we developed a FISH protocol that might be used directly in gastric human mucosa using a *H*. *pylori* specific LNA-based oligonucleotide probe that has an excellent performance at 37°C as demonstrated in our previous work. Therefore, we report the development of a FISH-based method that can be carried out at 37° C and at a large range of pH (2–7), using only ethanol as a fixative agent for 15 min, and a hybridization solution consisting of 0.9 M NaCl and 0.5 M urea. In this method, the washing step can be performed by the gastric mucus that is naturally present in the stomach. This final method proved to be non-toxic, while retaining the specificity and sensitivity towards *H*. *pylori*.

This promising new method can be used *in vivo* in future clinical applications, in combination with a confocal laser endomicroscope for *H*. *pylori* detection. It also provides a powerful new approach for the diagnosis of microorganisms in general. Future work will focus on using animal models as an *in vivo* proof-of-concept for FIVH.

## Supporting Information

S1 Fig A. IC-HPLC analysis of FAM HP_ LNA/2OMe _PS oligonucleotide probe. The spectrum from FAM HyP-PS without treatment shows similar retention time comparatively to the spectrum from FAM HP_ LNA/2OMe _PS oligonucleotide probe after treatment in a pH2 buffer and pH4 buffer. B. Mass spectrum of FAM HP_ LNA/2OMe _PS oligonucleotide obtained by MALDI-TOF. The spectrum from the probe without treatment is similar to the spectrum after treatment in a pH2 buffer and pH4 buffer.
(TIF)

S2 Fig Optimization of hybridization condition of HP_ LNA/2OMe _PS oligonucleotide probe in pure culture of *H. pylori* strain 26695 (ATCC 700392) at different types of pH. A. Optimization of washing step using a standard buffer or an aqueous buffer during 15 min. B. Optimization of hybridization time. Hybridizations steps are performed using a standard hybridization buffer and a washing step during 15 min with aqueous buffer. C. Use of different types of hybridization buffer at 30 minutes of hybridization. Buffer 1: 0.1% (v/v) Triton-X, 5 mM of EDTA disodium salt 2-hydrate, 4M urea and 900 mM NaCl. Buffer 2: 0.1% (v/v) Triton-X, 5 mM of EDTA disodium salt 2-hydrate and 900 mM NaCl. Buffer 3: 4M urea and 900 mM NaCl. Buffer 4: 2M urea and 900 mM NaCl. Buffer 5: 0.5M urea and 900 mM NaCl. D. Optimization of permeabilization step. Hybridization step was performed using 0.5M urea and 900 mM NaCl during 30 min. The washing step used in these experiments was with aqueous buffer during 15 min.
(TIF)

S3 Fig Detection of *H. pylori* using the FAM HP_ LNA/2OMe _PS oligonucleotide probe, without permeabilization. Smear of pure culture of *H*. *pylori* strain 26695 (ATCC 700392) observed by epifluorescent microscopy. A-C. Experiment using 200 nM of HyP_PS probe. D-F. Smears without probe were used as negative control. All images were taken at equal exposure times. Original magnification: 1000x. Scale bar = 10 μm.
(TIF)

S4 Fig Detection of *H. pylori* using the red fluorescent HP_ LNA/2OMe _PS oligonucleotide probe. A-F Smear of pure culture of *H*. *pylori* strain 26695 (ATCC 700392) observed by epifluorescent microscopy. A-C. Experiment using 200 nM of HP_ LNA/2OMe _PS oligonucleotide probe. D-F. Smears without probe were used as negative control. All images were taken at equal exposure times. Original magnification: 1000x. G-I. Relative fluorescence histograms of LNA-FISH targeting *H*. *pylori* in different pH for two different assays—Blue: negative control with no probe; Red: positive sample. Scale bar = 5 μm.
(TIF)

S5 Fig Detection of *H. pylori* at pH1 using gastric simulated juice by FAM HP_ LNA/2OMe _PS and Cy3 HP_ LNA/2OMe _PS oligonucleotide probes. A. FAM HP_ LNA/2OMe _PS oligonucleotide probe. B. Cy3 HP_ LNA/2OMe _PS oligonucleotide probe. C. negative control.
(TIF)

S1 Table Experimental design matrix and corresponding observed results of arbitrary fluorescence intensity (AUF). (DOCX)

S2 Table Analysis of variance (ANOVA) for linear model. The adequacy of the model was checked using analysis of variance.
(DOCX)
