# Supplementary figures and images for: Towards Fluorescence In Vivo Hybridization (FIVH) Detection of H. pylori in Gastric Mucosa Using Advanced LNA Probes
Source: PLoS One. 2015 Apr 27;10(4):e0125494. doi: 10.1371/journal.pone.0125494 (PMC4410960; doi:10.1371/journal.pone.0125494)

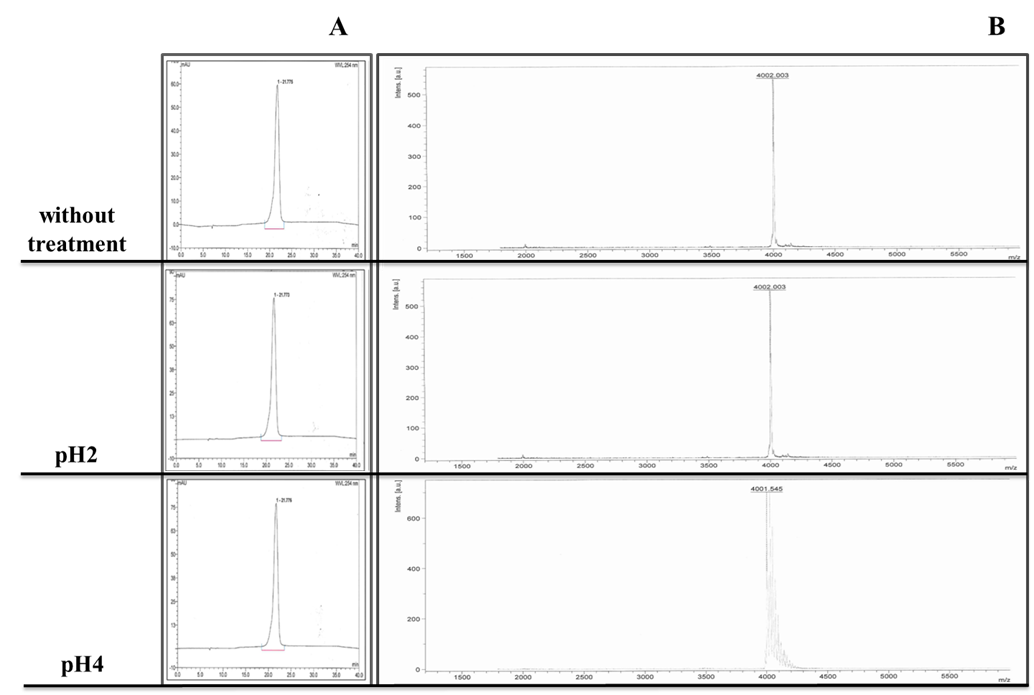

Supplement: S1 Fig — The spectrum from FAM HyP-PS without treatment shows similar retention time comparatively to the spectrum from FAM HP_ LNA/2OMe _PS oligonucleotide probe after treatment in a pH2 buffer and pH4 buffer. B. Mass spectrum of FAM HP_ LNA/2OMe _PS oligonucleotide obtained by MALDI-TOF. The spectrum from the probe without treatment is similar to the spectrum after treatment in a pH2 buffer and pH4 buffer. (TIF) [file pone.0125494.s001.tif]

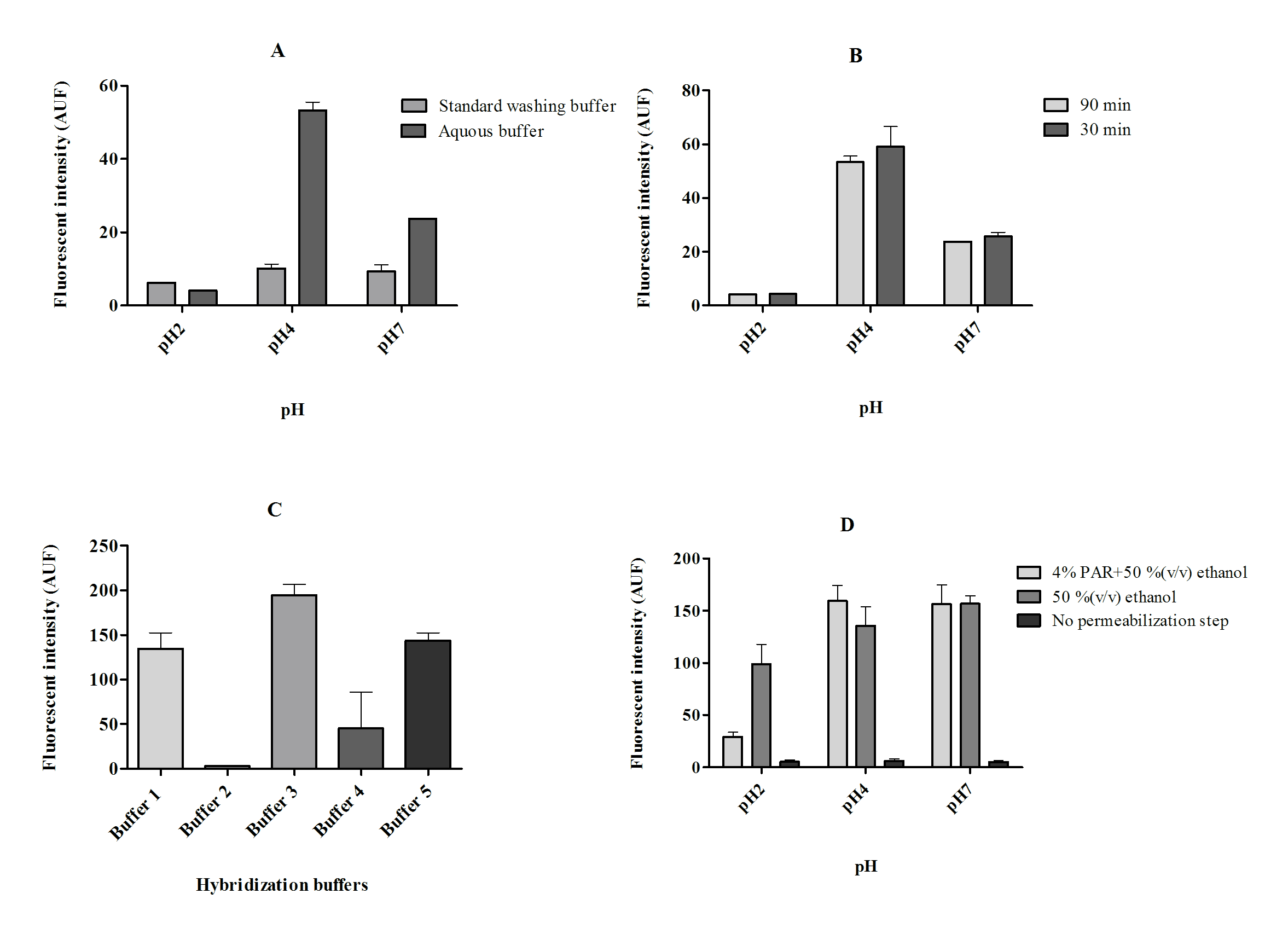

Supplement: S2 Fig — A. Optimization of washing step using a standard buffer or an aqueous buffer during 15 min. B. Optimization of hybridization time. Hybridizations steps are performed using a standard hybridization buffer and a washing step during 15 min with aqueous buffer. C. Use of different types of hybridization buffer at 30 minutes of hybridization. Buffer 1: 0.1% (v/v) Triton-X, 5 mM of EDTA disodium salt 2-hydrate, 4M urea and 900 mM NaCl. Buffer 2: 0.1% (v/v) Triton-X, 5 mM of EDTA disodium salt 2-hydrate and 900 mM NaCl. Buffer 3: 4M urea and 900 mM NaCl. Buffer 4: 2M urea and 900 mM NaCl. Buffer 5: 0.5M urea and 900 mM NaCl. D. Optimization of permeabilization step. Hybridization step was performed using 0.5M urea and 900 mM NaCl during 30 min. The washing step used in these experiments was with aqueous buffer during 15 min. (TIF) [file pone.0125494.s002.tif]

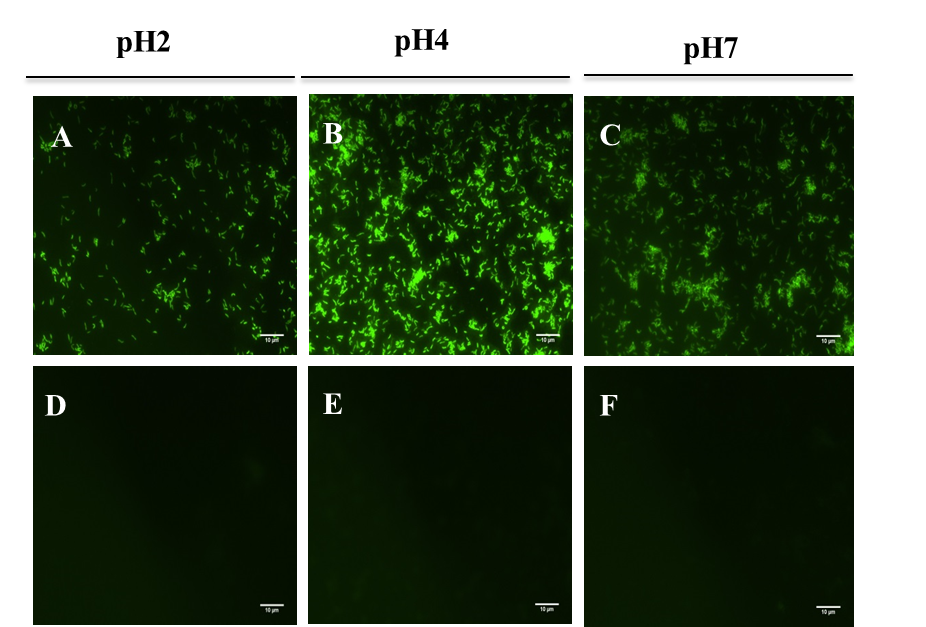

Supplement: S3 Fig — Smear of pure culture of H. pylori strain 26695 (ATCC 700392) observed by epifluorescent microscopy. A-C. Experiment using 200 nM of HyP_PS probe. D-F. Smears without probe were used as negative control. All images were taken at equal exposure times. Original magnification: 1000x. Scale bar = 10 μm. (TIF) [file pone.0125494.s003.tif]

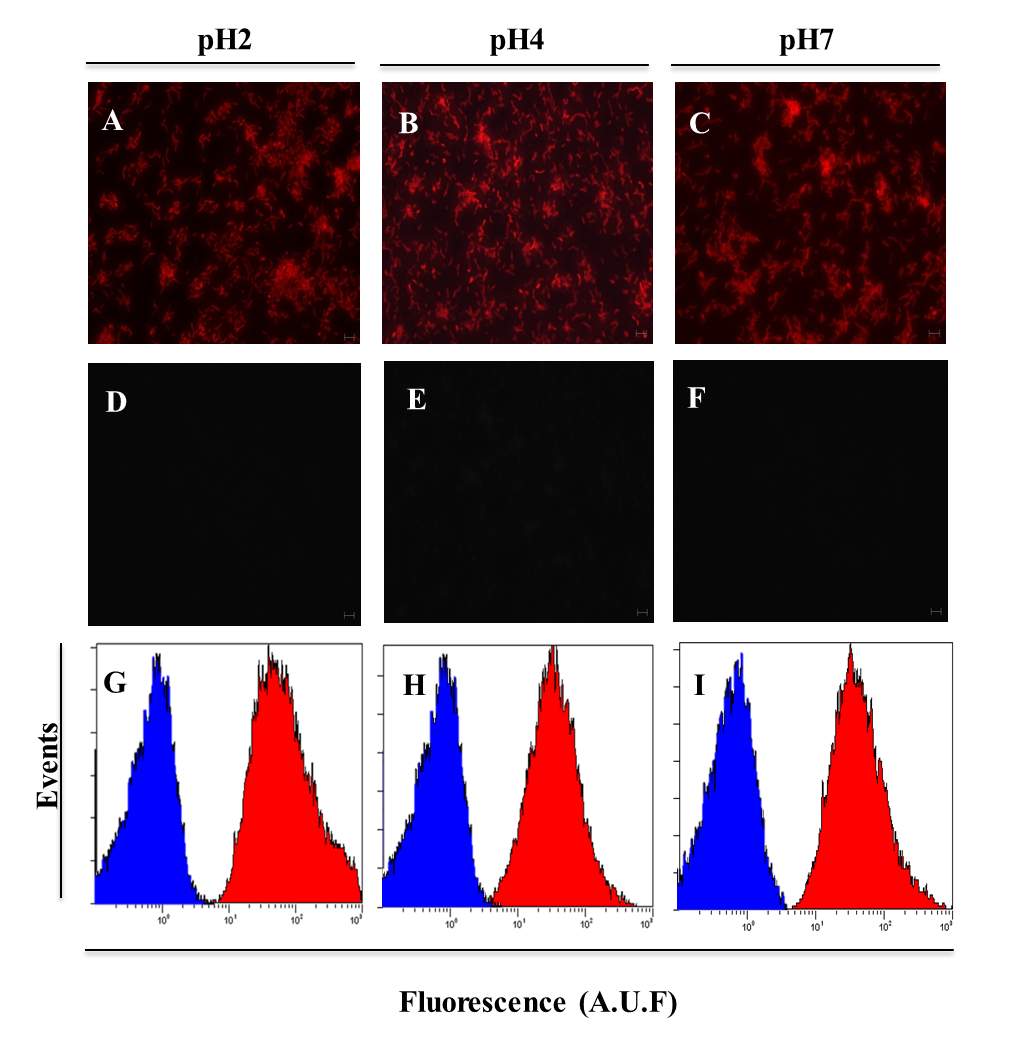

Supplement: S4 Fig — A-F Smear of pure culture of H. pylori strain 26695 (ATCC 700392) observed by epifluorescent microscopy. A-C. Experiment using 200 nM of HP_ LNA/2OMe _PS oligonucleotide probe. D-F. Smears without probe were used as negative control. All images were taken at equal exposure times. Original magnification: 1000x. G-I. Relative fluorescence histograms of LNA-FISH targeting H. pylori in different pH for two different assays—Blue: negative control with no probe; Red: positive sample. Scale bar = 5 μm. (TIF) [file pone.0125494.s004.tif]

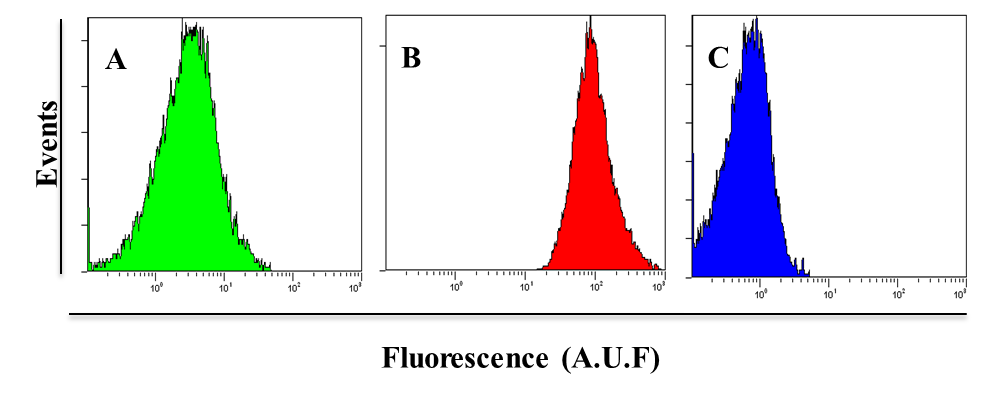

Supplement: S5 Fig — A. FAM HP_ LNA/2OMe _PS oligonucleotide probe. B. Cy3 HP_ LNA/2OMe _PS oligonucleotide probe. C. negative control. (TIF) [file pone.0125494.s005.tif]
