# Supplementary material for: Towards Fluorescence In Vivo Hybridization (FIVH) Detection of H. pylori in Gastric Mucosa Using Advanced LNA Probes
Source: PLoS One. 2015 Apr 27;10(4):e0125494. doi: 10.1371/journal.pone.0125494 (PMC4410960; doi:10.1371/journal.pone.0125494)
Supplement: S1 Table — (DOCX) [file pone.0125494.s006.docx]

| **Run** | **Factor 1: Time** | **Factor 2: pH** | **Response: Fluorescence intensity (AFU)** |
| --- | --- | --- | --- |
| 1 | 52.75 | 4.50 | 898.00 |
| 2 | 15.50 | 2.00 | 613.00 |
| 3 | 52.75 | 4.50 | 1064.00 |
| 4 | 90.00 | 7.00 | 329.76 |
| 5 | 15.50 | 7.00 | 464.58 |
| 6 | 52.75 | 4.50 | 984.30 |
| 7 | 90.00 | 2.00 | 815.96 |
| 8 | 52.75 | 0.96 | 2347.78 |
| 9 | 52.75 | 8.04 | 90.49 |
| 10 | 52.75 | 4.50 | 217.60 |
| 11 | 0.07 | 4.50 | 80.00 |
| 12 | 105.43 | 4.50 | 344.26 |
| 13 | 52.75 | 4.50 | 215.77 |
| 14 | 52.75 | 4.50 | 227.74 |
