# Supplementary material for: Towards Fluorescence In Vivo Hybridization (FIVH) Detection of H. pylori in Gastric Mucosa Using Advanced LNA Probes
Source: PLoS One. 2015 Apr 27;10(4):e0125494. doi: 10.1371/journal.pone.0125494 (PMC4410960; doi:10.1371/journal.pone.0125494)
Supplement: S2 Table — The adequacy of the model was checked using analysis of variance. (DOCX) [file pone.0125494.s007.docx]

| **Model** | **Sequential**  **p-value** | **Lack of fit**  **p-value** | **R-square** |
| --- | --- | --- | --- |
| **Linear** | 0.0178 | 0.0001 | 0.5543 |
